# Supplementary material for: SIRT1 accelerates the progression of activity-based anorexia
Source: Nat Commun. 2020 Jun 4;11:2814. doi: 10.1038/s41467-020-16348-9 (PMC7272424; doi:10.1038/s41467-020-16348-9)
Supplement: Supplementary file 1 — Supplementary Information [file 41467_2020_16348_MOESM1_ESM.pdf]

Supplementary Information

SIRT1 accelerates the progression of activity-based anorexia

Robinette *et al.*

Supplemental Figures

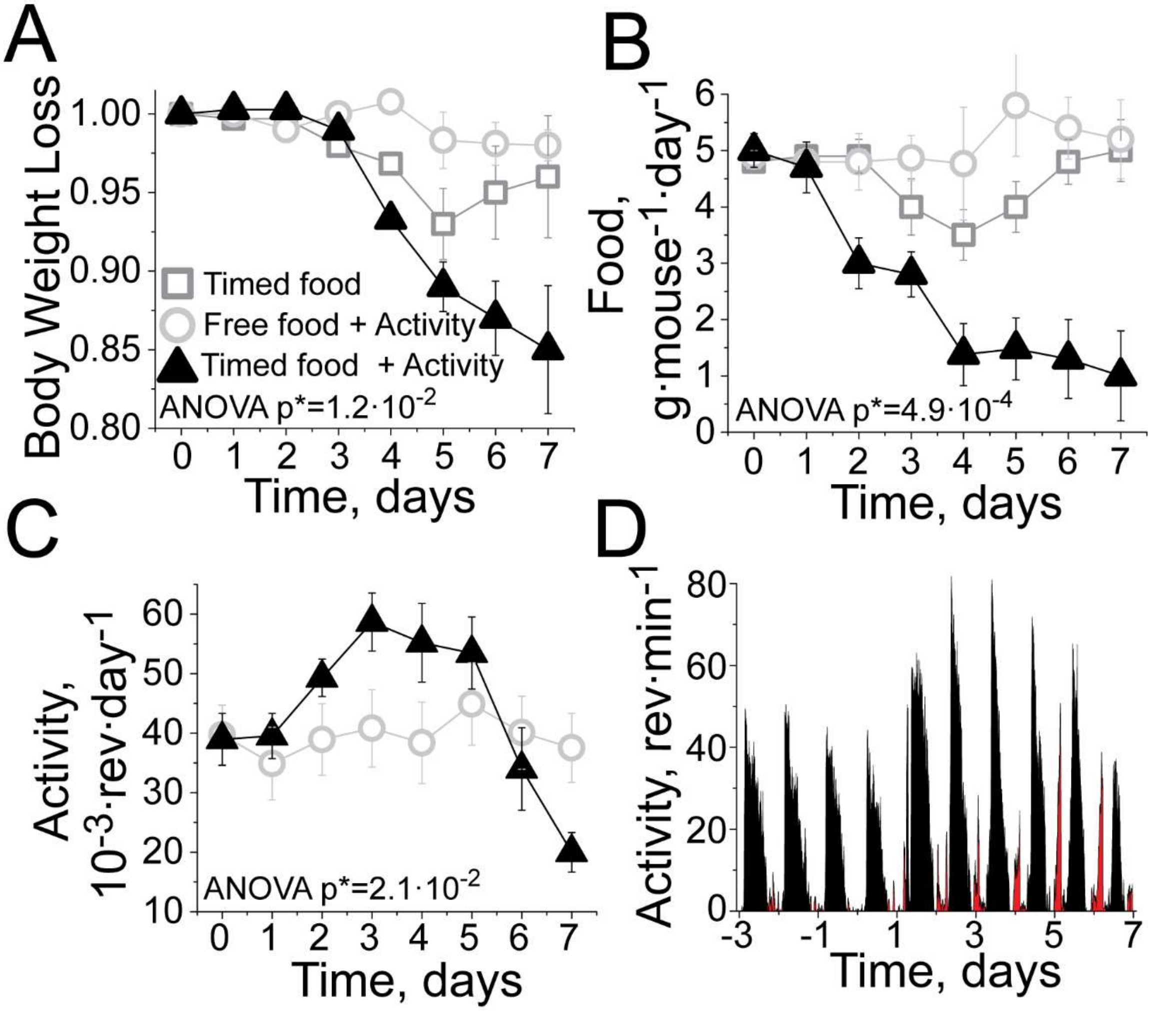

Supplementary Figure 1. Activity based anorexia (ABA) recapitulates many phenotypes of human AN. a) Rodents with both access to a running wheel and intermittent access to food (triangle) lose weight compared to littermates on the identical feeding regime but with a locked running wheel (square), or with access to a running wheel but with free access to food (circle). **b)** Mice subjected to ABA voluntarily reduce their food intake; food consumption per mouse per day is graphed against time. **c)** Animals subjected to ABA paradoxically increase their activity; number of running wheel revolutions per day is plotted against time for mice on the ABA protocol vs controls. **d)** Circadian activity of animals on ABA is plotted against time. Nocturnal activity is depicted in black and diurnal activity is depicted in red. As ABA is progressing, an increase in physical activity and a distortion of the circadian rhythm is observed. Statistical analysis was completed using two-way ANOVA with SEM error bars, n = 6. Source data are provided as a Source Data file.

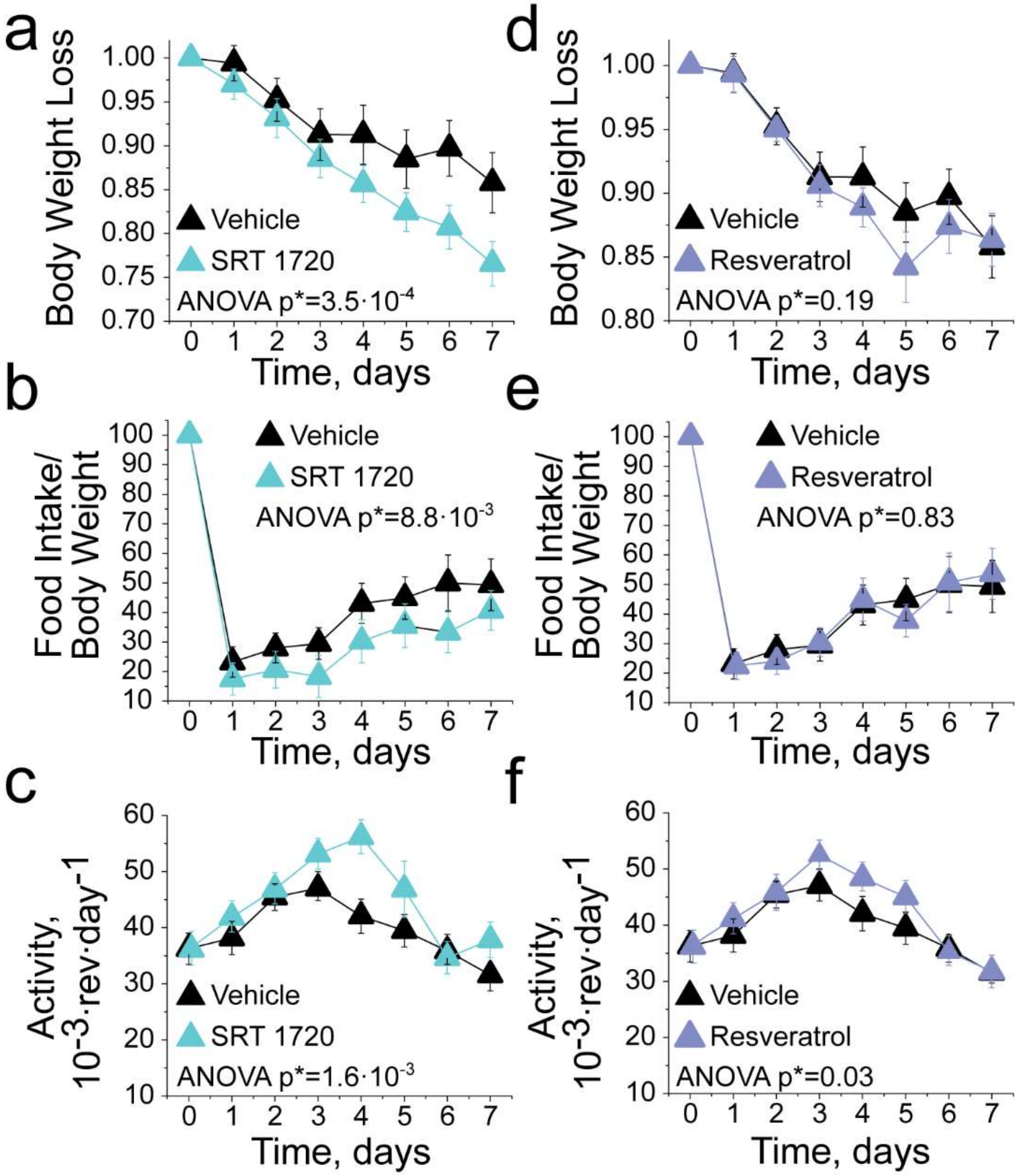

Supplementary Figure 2. Mice treated with SRT1720 are more susceptible to the ABA model while resveratrol has a null effect. a-c) SRT1720 treated mice (blue) lose weight more rapidly, decrease their food intake more severely, and are more hyperactive than their WT littermates (black). **d-f)** Resveratrol treated mice (purple) lose weight and decrease their food intake at about the same rate as their WT littermates. However, the mice injected with resveratrol do increase their activity more than the WT cohort. Statistical analysis was completed using two-way ANOVA with SEM error bars, n = 8. Source data are provided as a Source Data file.

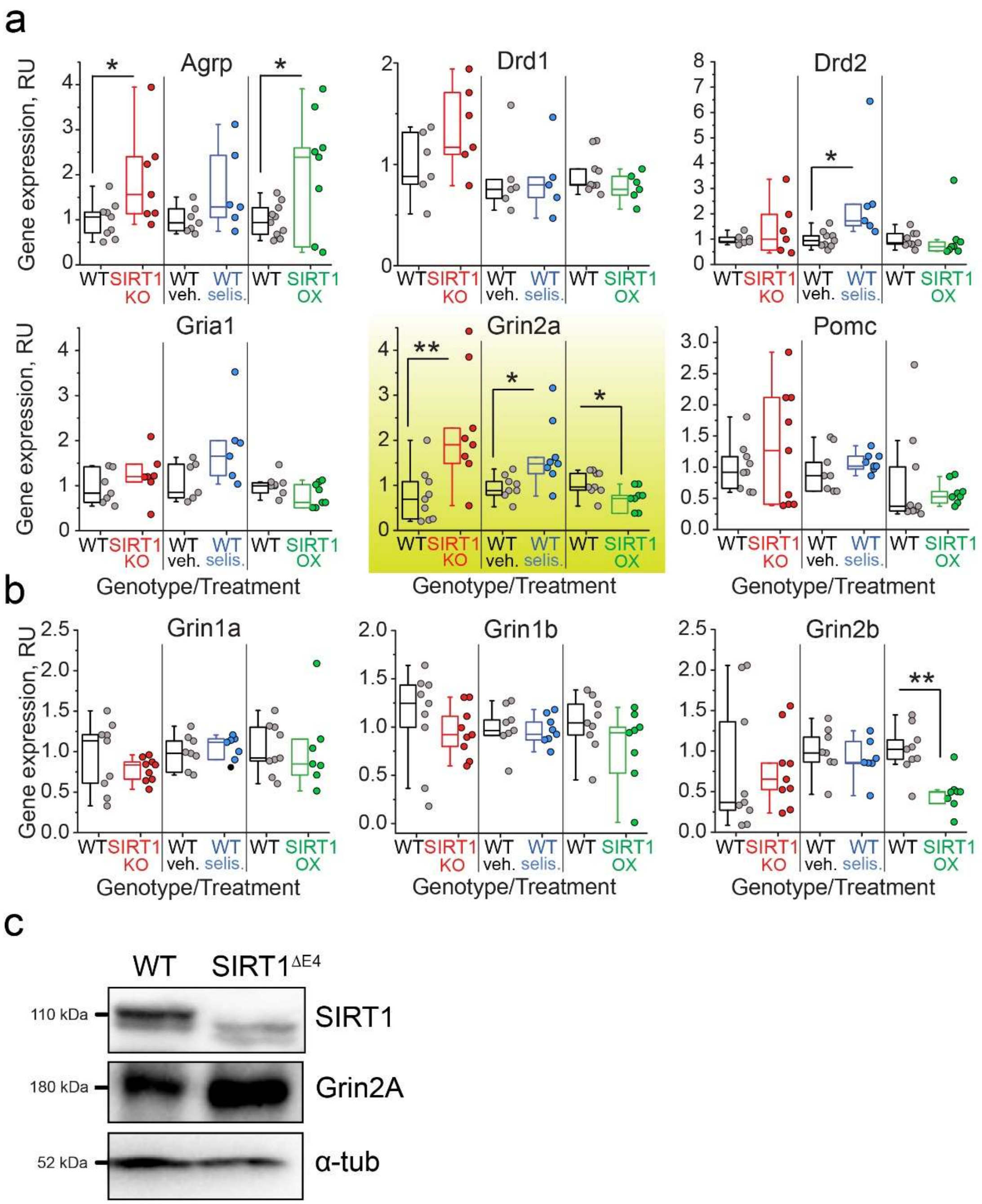

Supplementary Figure 3. SIRT1 represses Grin2A a) RT-PCR expression analysis of AGRP, Drd1, Drd2, GRIA1, Grin2a, and POMC in the cortex of mice that lack (red) or overexpress (green) SIRT1 in the brain, as well as those treated with SIRT1 inhibitor selisistat (blue) is shown. Grin2a is the only gene with significant and opposite changes in transcription levels, n ≥ 6. **b)** Expression analysis of three other NMDA receptor subunits, Grin1a, Grin1b, and Grin2b, n ≥ 6. **c)** Representative SDS-PAGE analysis of SIRT1 BSKO mice. Levels of Grin2A protein are elevated *in vivo* in BSKO mice. This is representative of three independent experiments. P-values were calculated using unpaired two-tailed t-tests, * $P < 0.05$, ** $P < 0.005$. The box-plots represent the median,

25th, and 75th percentiles of the data and the whiskers represent 5-95% of the data. Source data are provided as a Source Data file.

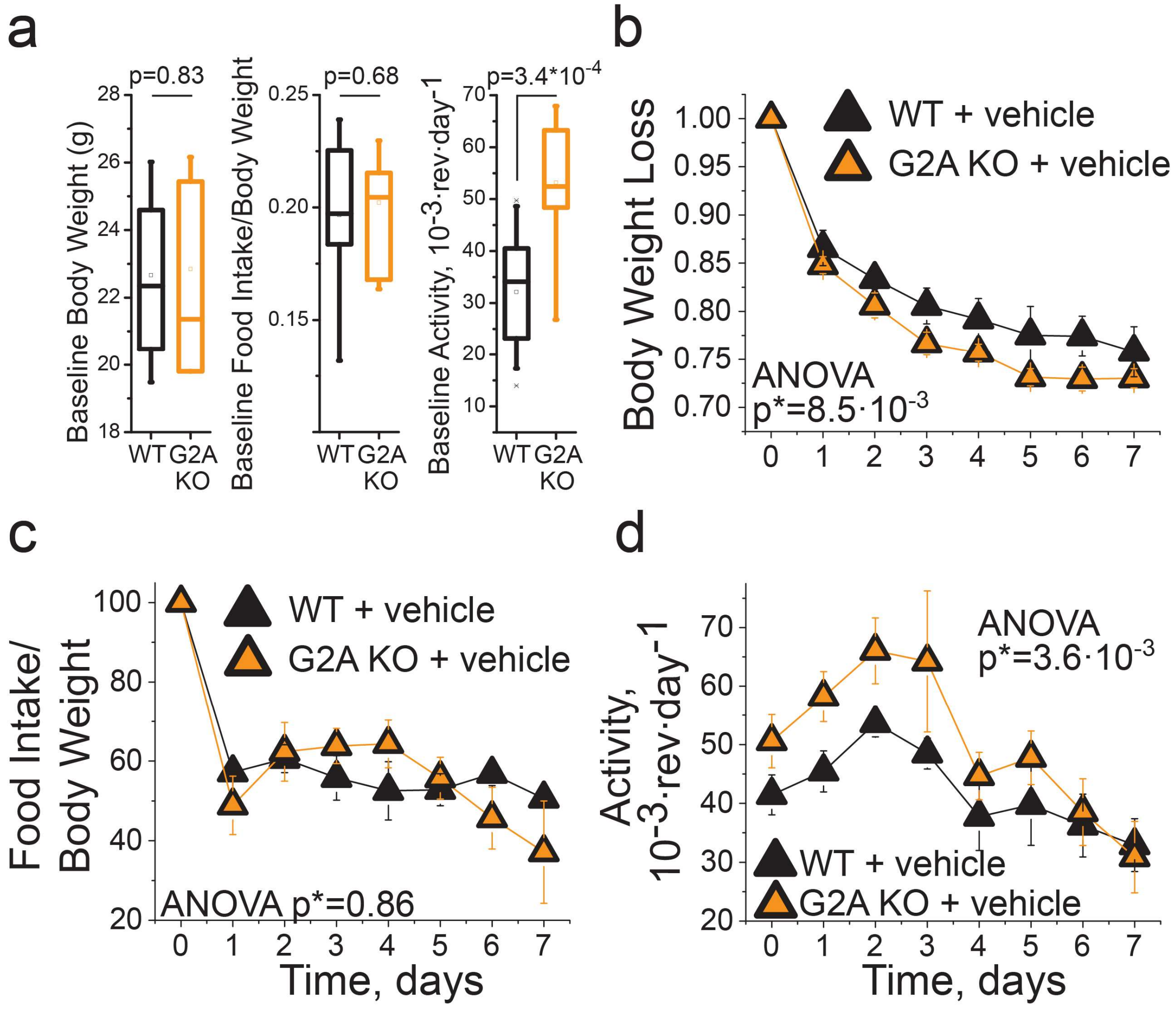

Supplementary Figure 4. G2A KO mice are more susceptible to the ABA model. a) G2A KO mice (orange) weight weigh and eat the same as their WT littermates (black). However, they are more active than the WT mice. **b-d)** The G2A KO mice increase their activity levels much higher than the WT cohort, likely leading to the more rapid loss of body weight. However, there was no difference between the WT and G2A KO mice when comparing their food intake. Statistical analysis was completed using two-way ANOVA with SEM error bars, n = 8. For the box plots, P-values were calculated using unpaired two-tailed t-tests. The box-plots represent the median, 25th, and 75th percentiles of the data and the whiskers represent 5-95% of the data. Source data are provided as a Source Data file.

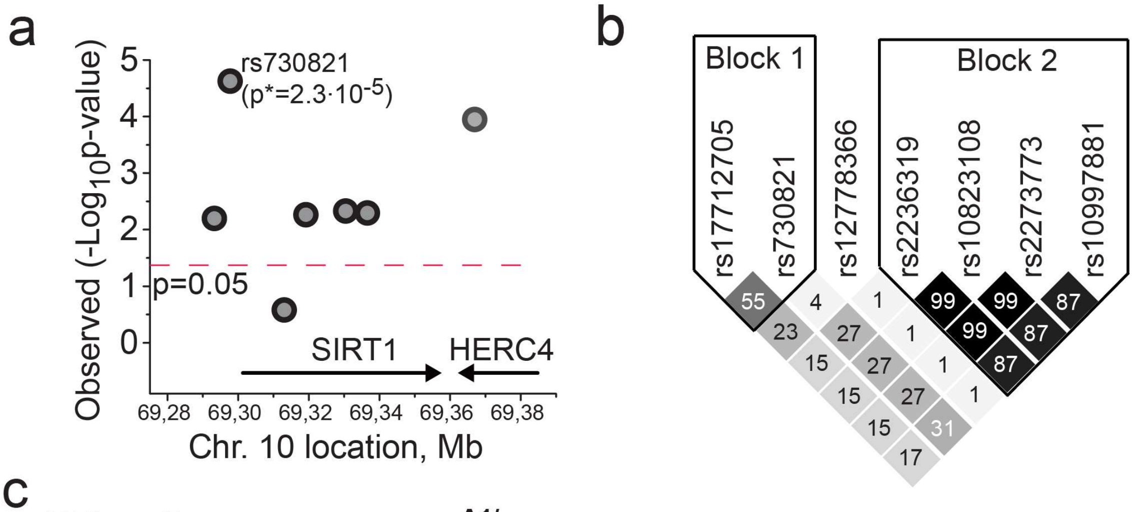

c

Study	AN Cases#/ Controls#	SNP ID	Chr.10 position	A1/ A2	Freq. (cases)	Freq. (cntrl)	OR	χ^2	P	Bonf.P	Permut.P
Philadelphia Study	1001/ 1987	rs17712705	69293277	A/G	0.3267	0.3624	1.171	7.447	0.006355	0.04449	0.02893
		rs730821	69297765	G/A	0.1998	0.249	1.329	17.85	**0.000023**	**0.00016**	**0.00015**
		rs12778366	69313085	C/T	0.128	0.118	0.910	1.243	0.26480	1	0.658
		rs2236319	69319226	G/A	0.0654	0.0859	1.356	7.711	0.005488	0.03842	0.02617
		rs10823108	69330500	A/G	0.0649	0.0858	1.347	7.989	0.004707	0.03295	0.02259
		rs2273773	69336604	C/T	0.0649	0.0856	1.347	7.841	0.005106	0.03574	0.02309
		rs10997881	69367001	C/T	0.066	0.0956	1.498	14.9	0.000113	0.00079	0.00062
Italian Study	112/ 3937	rs730821	69297765	G/A	0.1343	0.2436	2.082	13.72	**0.000212**	**0.000424**	**0.0011**
		rs10997881	69367001	C/T	0.0707	0.0841	1.193	0.477	0.4896	0.9792	0.7033

Supplementary Figure 5. SIRT1 gene variants are associated with susceptibility to AN in humans. a) The location and of the seven SNPs genotyped from human females with or without AN. **b)** Four C-terminus SNPs in SIRT1 are in strong linkage disequilibrium (LD). The values in the diamonds represents the R^2. **c)** Summary of the associations between the SNPs in SIRT1 and AN in humans, in two independent cohorts. A combination of R and p-link software was used to create a linear regression model, A1/A2 are the minor/major alleles, OR is the odds ratio. Bonferroni correction and 100,000 rounds of permutation analysis were used to account for multiple testing.

Supplementary Figure 6. Uncropped Original Scans

Figure 3c

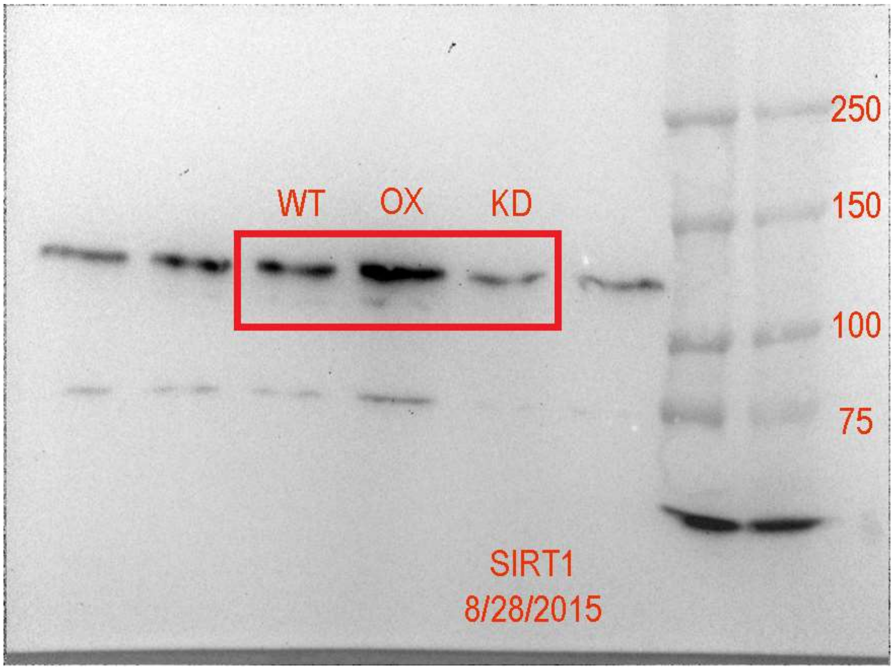

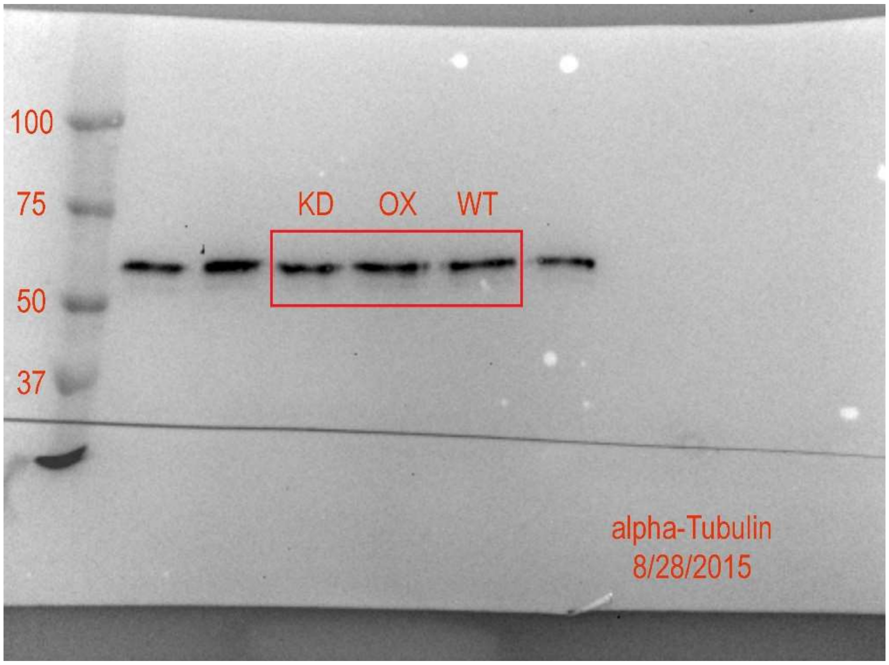

Figure 4c

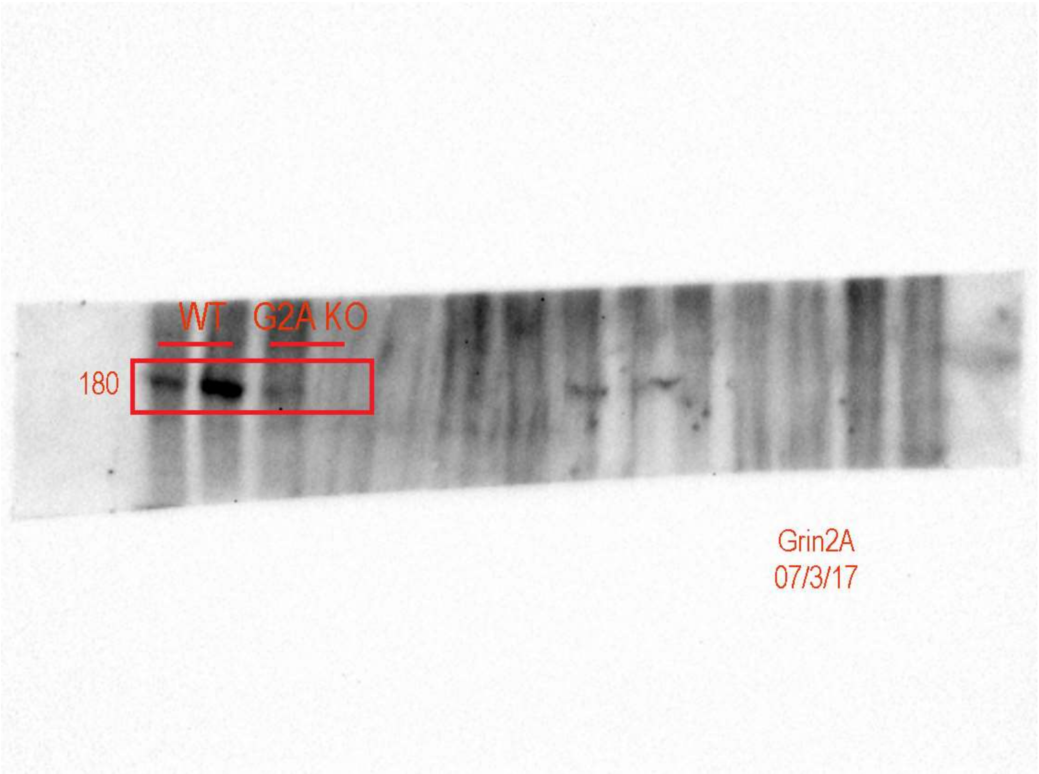
WT
G2A KO
180
Grin2A
07/3/17

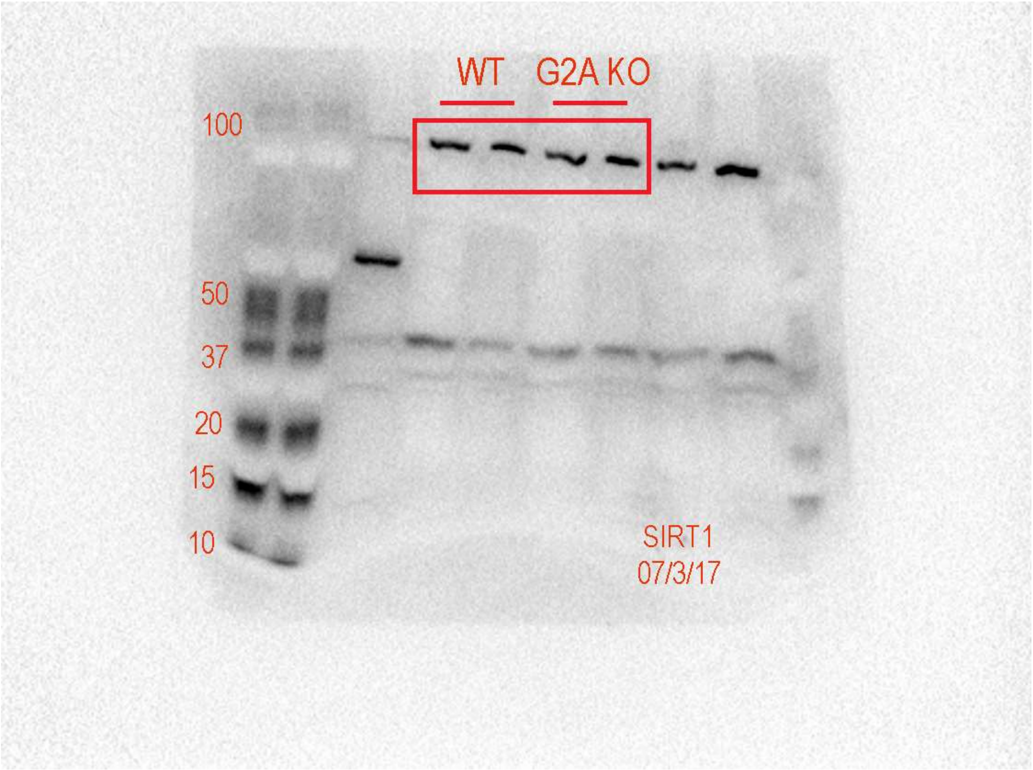
WT
G2A KO
100
50
37
20
15
10
SIRT1
07/3/17

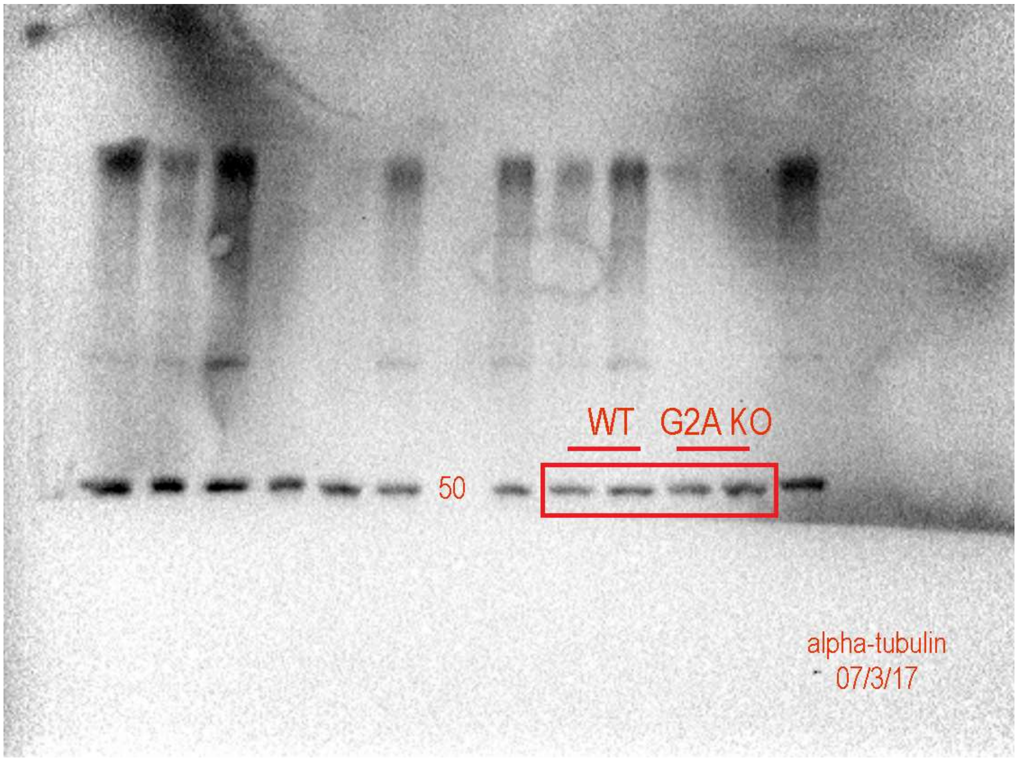

Supplemental Figure 2a

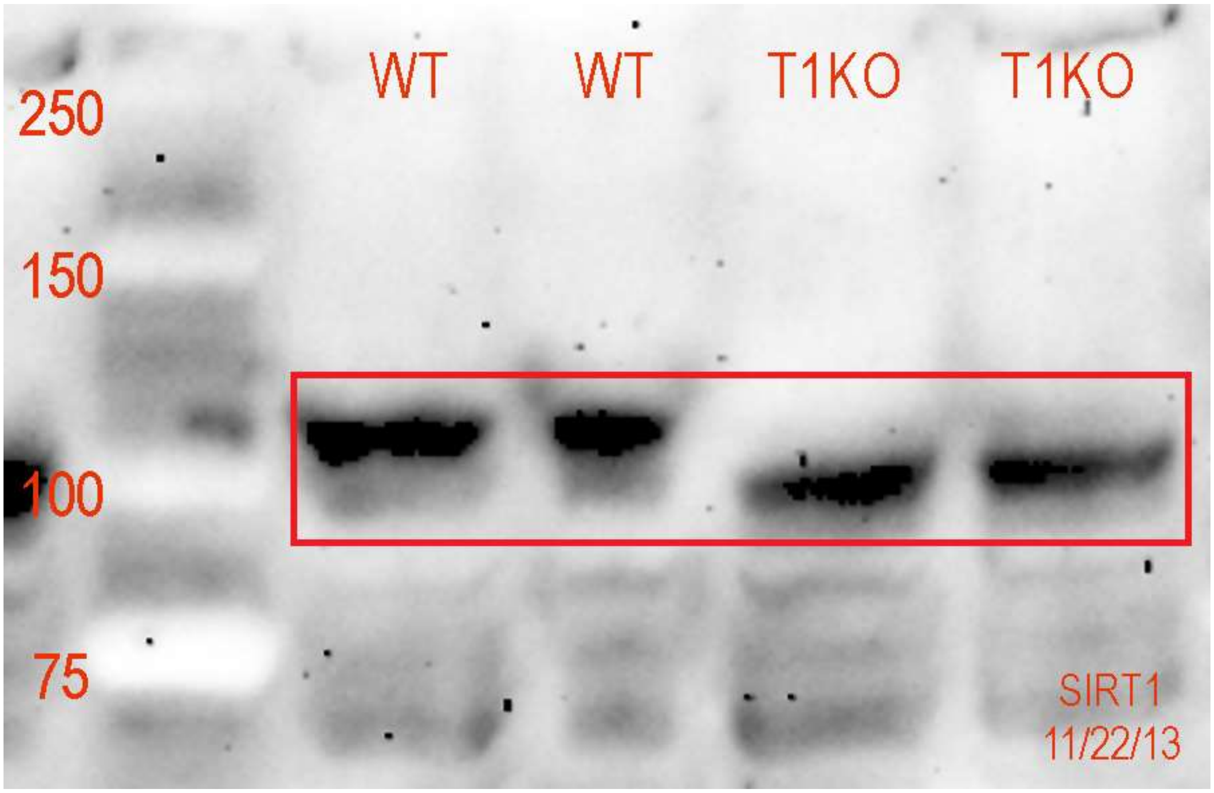

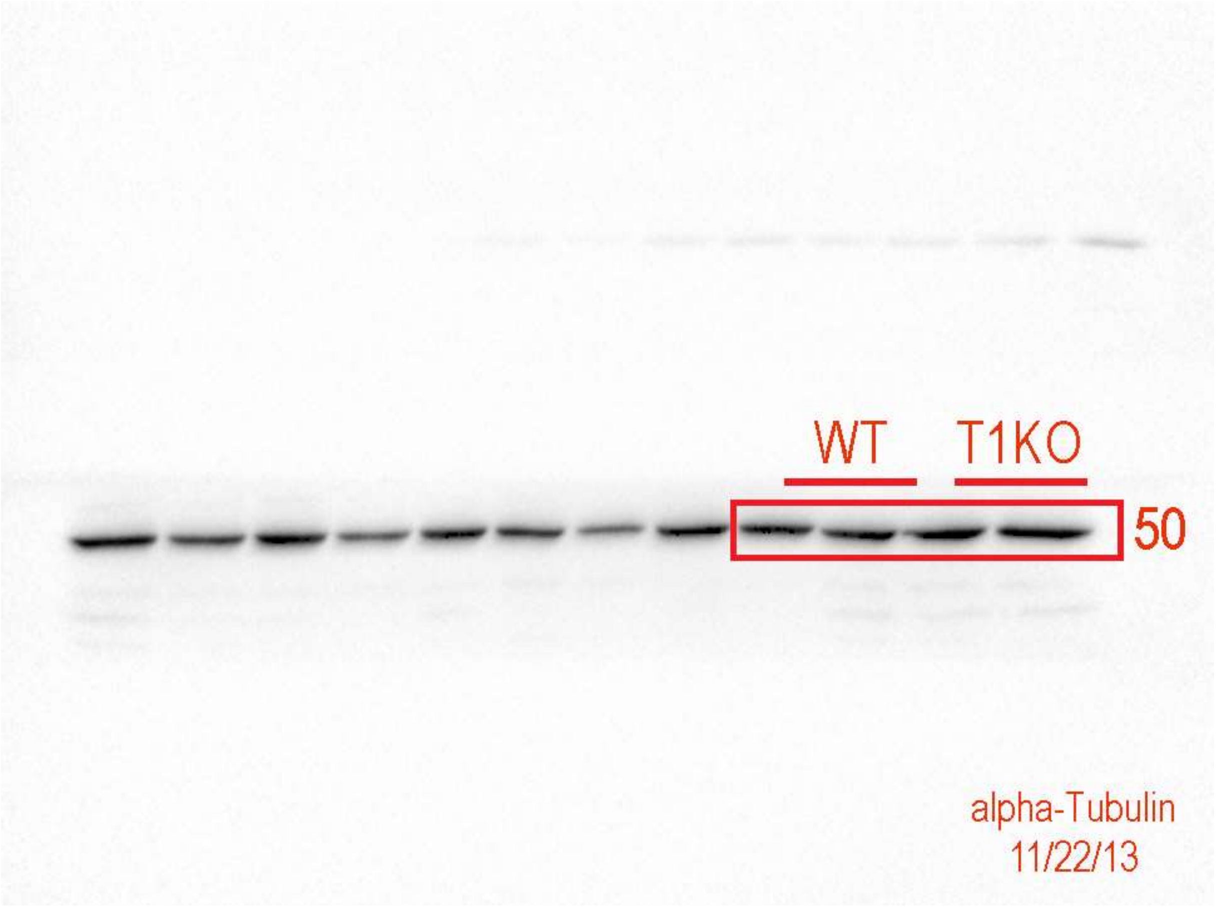
WT
T1KO
50
alpha-Tubulin
11/22/13

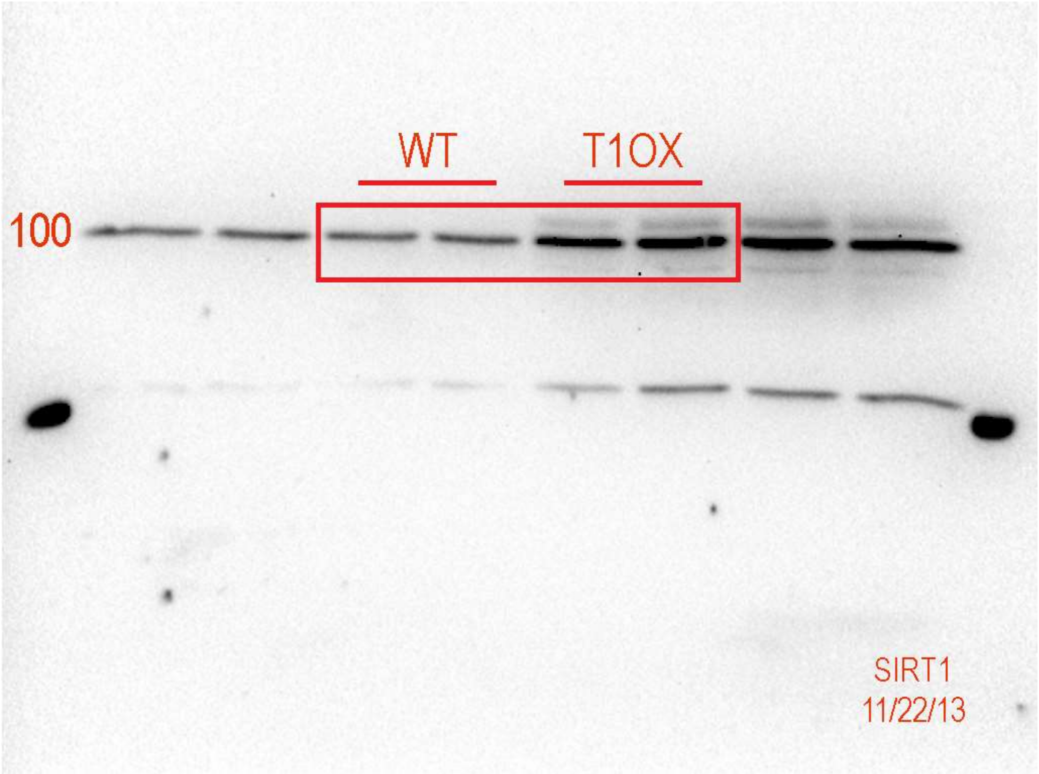
WT
T1OX
100
SIRT1
11/22/13

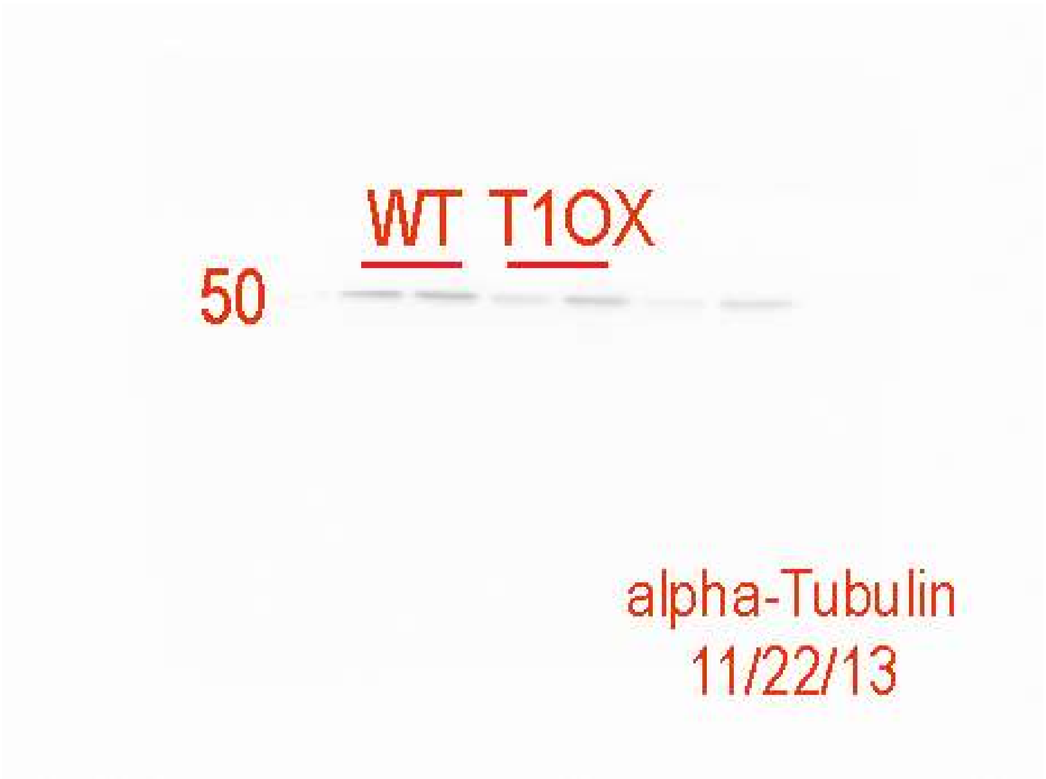

Supplemental Figure 4c

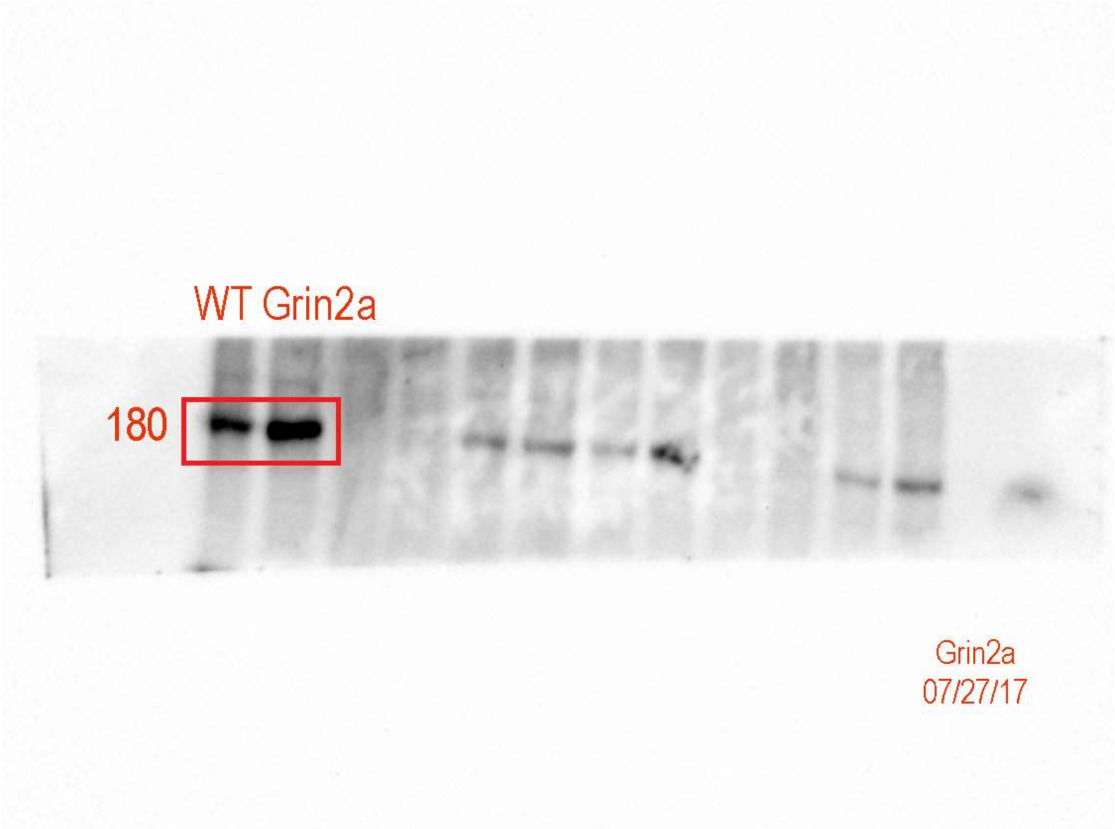

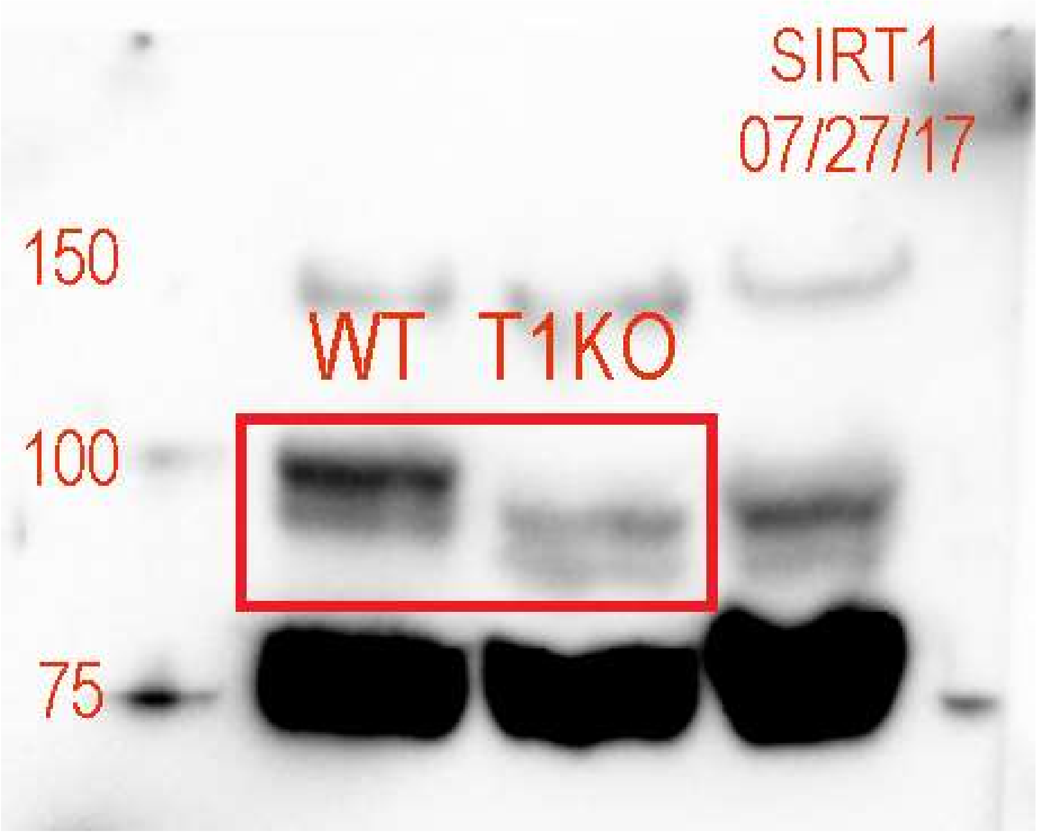
SIRT1
07/27/17
150
100
75
WT T1KO

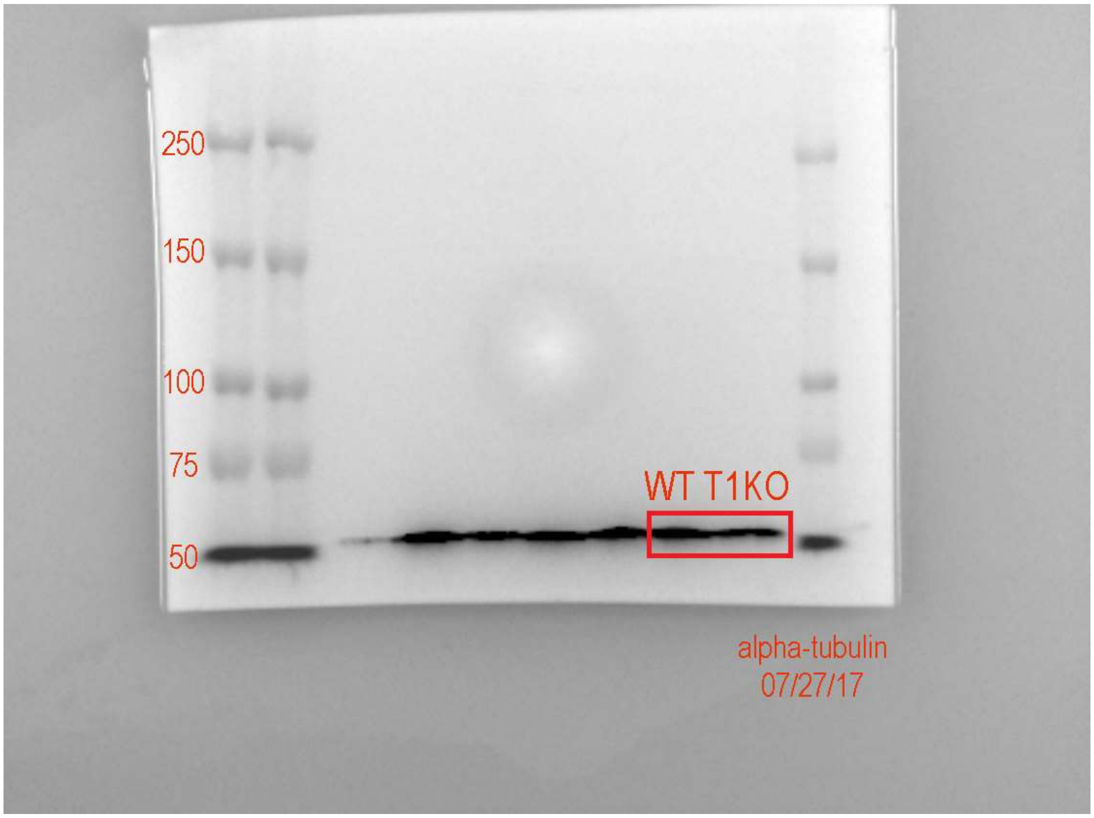
250
150
100
75
50
WT T1KO
alpha-tubulin
07/27/17
