# Supplementary material for: SIRT1 accelerates the progression of activity-based anorexia
Source: Nat Commun. 2020 Jun 4;11:2814. doi: 10.1038/s41467-020-16348-9 (PMC7272424; doi:10.1038/s41467-020-16348-9)
Supplement: Supplementary file 3 — Reporting Summary [file 41467_2020_16348_MOESM3_ESM.pdf]

nature research

Corresponding author(s): Sergiy Libert

Last updated by author(s): Mar 17, 2020

Reporting Summary

Nature Research wishes to improve the reproducibility of the work that we publish. This form provides structure for consistency and transparency in reporting. For further information on Nature Research policies, see Authors & Referees and the Editorial Policy Checklist.

Statistics

For all statistical analyses, confirm that the following items are present in the figure legend, table legend, main text, or Methods section.

n/a	Confirmed	
☐	☒	The exact sample size (*n*) for each experimental group/condition, given as a discrete number and unit of measurement
☐	☒	A statement on whether measurements were taken from distinct samples or whether the same sample was measured repeatedly
☐	☒	The statistical test(s) used AND whether they are one- or two-sided<br>*Only common tests should be described solely by name; describe more complex techniques in the Methods section.*
☐	☒	A description of all covariates tested
☐	☒	A description of any assumptions or corrections, such as tests of normality and adjustment for multiple comparisons
☐	☒	A full description of the statistical parameters including central tendency (e.g. means) or other basic estimates (e.g. regression coefficient) AND variation (e.g. standard deviation) or associated estimates of uncertainty (e.g. confidence intervals)
☐	☒	For null hypothesis testing, the test statistic (e.g. *F*, *t*, *r*) with confidence intervals, effect sizes, degrees of freedom and *P* value noted<br>*Give P values as exact values whenever suitable.*
☒	☐	For Bayesian analysis, information on the choice of priors and Markov chain Monte Carlo settings
☐	☒	For hierarchical and complex designs, identification of the appropriate level for tests and full reporting of outcomes
☐	☒	Estimates of effect sizes (e.g. Cohen's *d*, Pearson's *r*), indicating how they were calculated

Our web collection on statistics for biologists contains articles on many of the points above.

Software and code

Policy information about availability of computer code

Data collection	No software was used that has not been described in published literature.
Data analysis	Microsoft Excel 2016, Origin Pro 8.0, and R version 3.2.5 were used for analysis

For manuscripts utilizing custom algorithms or software that are central to the research but not yet described in published literature, software must be made available to editors/reviewers. We strongly encourage code deposition in a community repository (e.g. GitHub). See the Nature Research guidelines for submitting code & software for further information.

Data

Policy information about availability of data

All manuscripts must include a data availability statement. This statement should provide the following information, where applicable:

- Accession codes, unique identifiers, or web links for publicly available datasets
- A list of figures that have associated raw data
- A description of any restrictions on data availability

The source data underlying Figs 1a-d, 2a-i, 3a-f, 4a-f, and Supplementary Figs. 1a-d, 2a-f, 3a-c, 4a-d are provided as a Source Data file. Data from dbGaP (https://www.ncbi.nlm.nih.gov/gap/) and Disgenet (https://www.disgenet.org/) were used in this study. All data supporting this study are included in this article and its supplementary information files, with raw data available from the corresponding author upon request.

Field-specific reporting

Please select the one below that is the best fit for your research. If you are not sure, read the appropriate sections before making your selection.

☒ Life sciences ☐ Behavioural & social sciences ☐ Ecological, evolutionary & environmental sciences

For a reference copy of the document with all sections, see nature.com/documents/nr-reporting-summary-flat.pdf

Life sciences study design

All studies must disclose on these points even when the disclosure is negative.

Sample size	Based on variability of prior collected data, necessary sample sizes were estimated using power analysis and reported on figures and in the text.
Data exclusions	No data was excluded.
Replication	All tissue and cell experiments were replicated at least three times. All animal experiments we completed with at least six mice that were entrained to eliminate the influence of stress and anxiety.
Randomization	The Neuro-2a cells were pooled before being split into experimental groups. Animals were assigned to experimental groups randomly.
Blinding	The investigators were not blinded during experiments or analysis. The setup of the lab was such that the experiment setup, the experiment, and the analysis was completed by the same person

Reporting for specific materials, systems and methods

We require information from authors about some types of materials, experimental systems and methods used in many studies. Here, indicate whether each material, system or method listed is relevant to your study. If you are not sure if a list item applies to your research, read the appropriate section before selecting a response.

Materials & experimental systems

n/a	Involved in the study	
☐	☒	Antibodies
☐	☒	Eukaryotic cell lines
☒	☐	Palaeontology
☐	☒	Animals and other organisms
☒	☐	Human research participants
☒	☐	Clinical data

Methods

n/a	Involved in the study	
☒	☐	ChIP-seq
☒	☐	Flow cytometry
☒	☐	MRI-based neuroimaging

Antibodies

Antibodies used	SIRT1 (1:1000) (Millipore 04-1557), α-Tubulin (1:5000) (ABCam ab7291), or GluN2a (1:1000) (Cell Signaling 4205) antibodies
Validation	The SIRT1 mouse monoclonal antibody has been validated in WB, IP, ICC, IHC, and ChIP by Millipore Sigma (https://www.emdmillipore.com/US/en/product/Anti-SIRT1-Antibody-clone-10E4,MM_NF-04-1557#). We additionally validated this antibody using known SIRT1 KO tissues. The α-Tubulin mouse monoclonal antibody has been validated in WB, Flow Cytometry, ICC, IF, IHC-P by Abcam (https://www.abcam.com/alpha-tubulin-antibody-dm1a-loading-control-ab7291.html). The GluN2a rabbit polyclonal antibody has been validated in WB by Cell Signaling Technology (https://www.cellsignal.com/products/primary-antibodies/nmda-receptor-2a-glun2a-antibody/4205).

Eukaryotic cell lines

Policy information about cell lines

Cell line source(s)	American Type Culture Collection (ATCC)
Authentication	Neuro-2a cells were validated by morphology, and origin-confirmed via species-specific RT-PCR.
Mycoplasma contamination	The cells routinely tested negative for mycoplasma contamination.
Commonly misidentified lines (See ICLAC register)	No commonly misidentified cell lines were used in the study.

Animals and other organisms

Policy information about studies involving animals; ARRIVE guidelines recommended for reporting animal research

Laboratory animals	This study used multiple strains of both male and female three-month-old mice including C57BL/6, SIRT1 BSKO and BSOX, nestin-cre (JAX Stock# 003771), and Grin2A KO mice. Animals were housed under specific pathogen free conditions with free-water and free-food (except when experimental conditions necessitated timed-food) supply with a 12 hours light/dark cycle.
Wild animals	This study did not involve wild animals.
Field-collected samples	This study did not involve samples collected in the field.
Ethics oversight	Institutional Animal Care and Use Committee (IACUC) of Cornell University

Note that full information on the approval of the study protocol must also be provided in the manuscript.
